# Supplementary material for: Cleavage site-directed antibodies reveal the prion protein in humans is shed by ADAM10 at Y226 and associates with misfolded protein deposits in neurodegenerative diseases
Source: Acta Neuropathol. 2024 Jul 9;148(1):2. doi: 10.1007/s00401-024-02763-5 (PMC11233397; doi:10.1007/s00401-024-02763-5)
Supplement: Supplementary file 1 — Supplementary file1 (PDF 4776 KB) [file 401_2024_2763_MOESM1_ESM.pdf]

Supplementary figures (and respective figure legends)

for Song, Kovac, Mohammadi et al.:

Cleavage site-directed antibodies reveal the prion protein in humans is shed by ADAM10 at Y226 and associates with misfolded protein deposits in neurodegenerative diseases

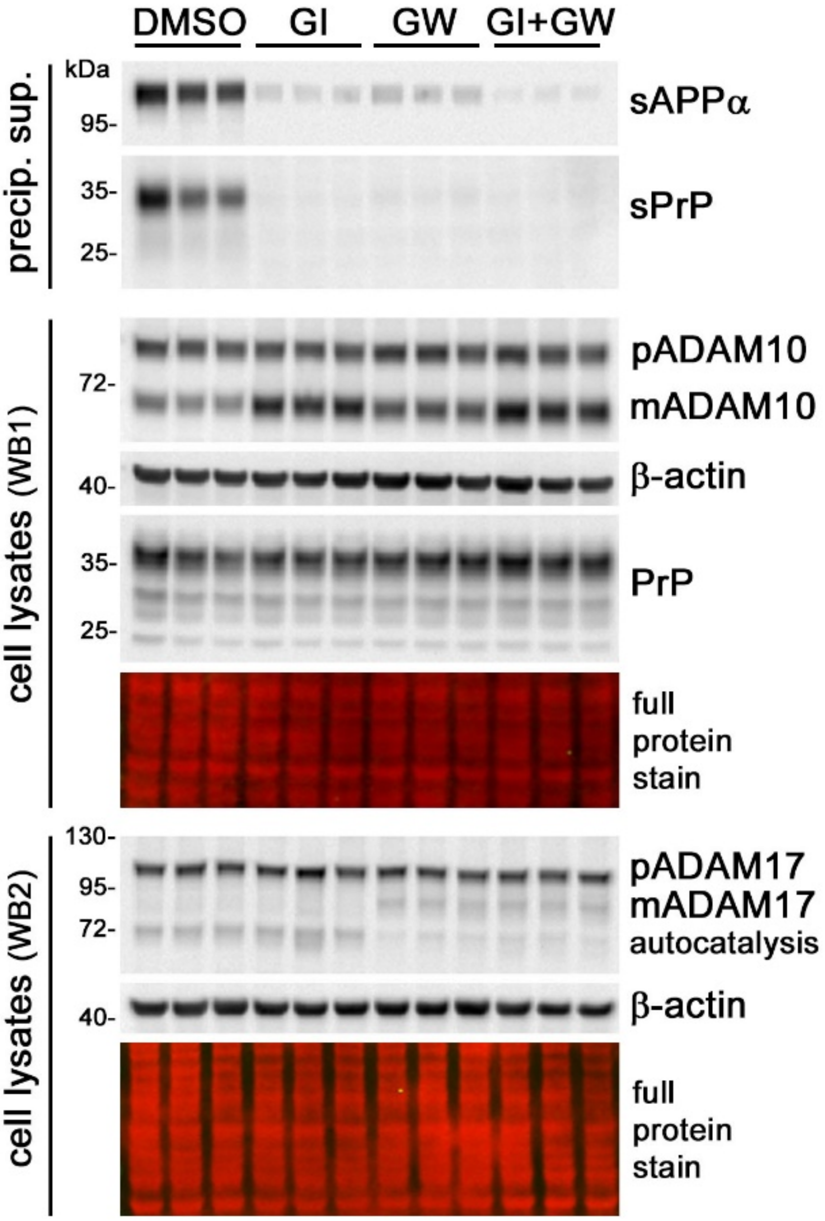

Supplementary Figure 1 (.jpg) WB analysis of sPrP and sAPPα (in TCA-precipitated conditioned media) and PrP, premature (p) and mature/active (m) ADAM10 (WB1) and ADAM17 (WB2) in lysates of the human glioblastoma-derived cell line U373-MG. Cells were treated with metalloprotease inhibitors GI254023X (GI) or/and GW280264X (GW) or with the diluent only (DMSO; as control). β-actin and total protein staining served as loading controls. Note that, as in A549 cells (Fig. 1c), GI alone does not inhibit ADAM17 activity (as judged by the lack of inhibition of a previously reported postlysis autocatalytic processing step [111]), whereas its inhibitory effect on ADAM10 is sufficient to abolish PrP shedding

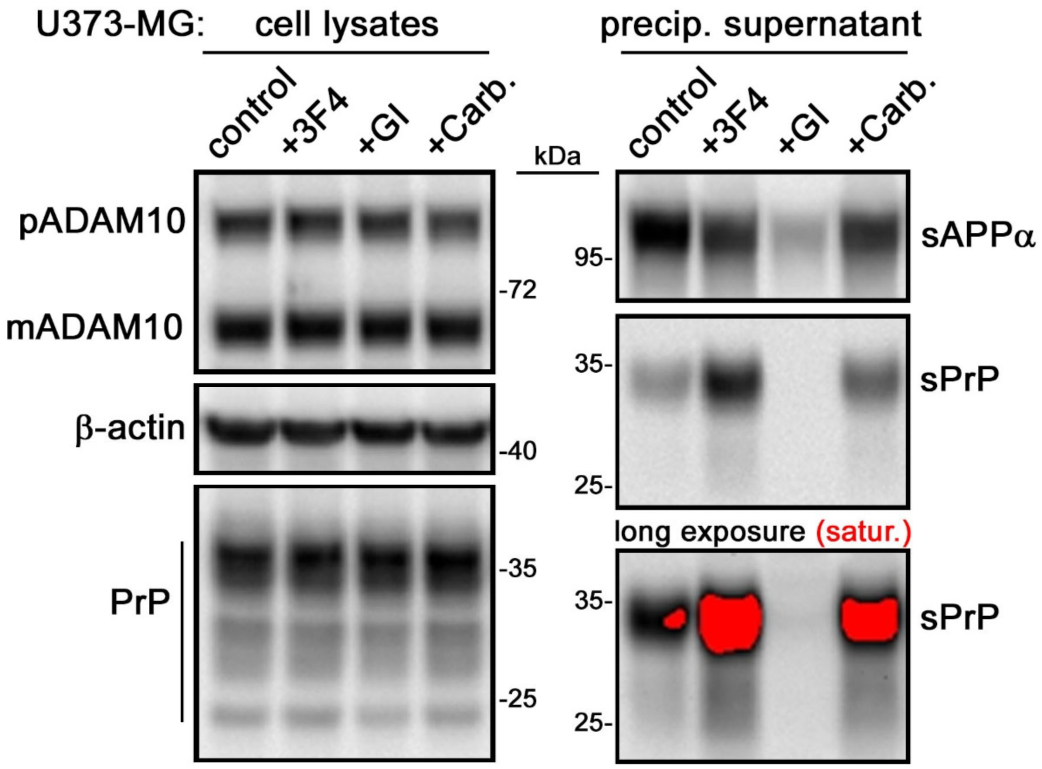

Supplementary Figure 2 (.jpg) WB analysis of human U373-MG cell lysates (blots on the left) and respective precipitated conditioned media supernatants (on the right). Cells were treated o.N. with the ADAM10 inhibitor GI or with either PrP-directed IgG (+3F4) or the compound Carbachol (+Carb.) to stimulate PrP shedding. While GI treatment only reduced sAPPα levels (likely due to residual ADAM17 activity compensating as alternative APP α-secretase for inhibition of ADAM10), it completely abolishes PrP shedding. Red signal indicates saturation upon long exposure densitometric detection of the sPrP blot

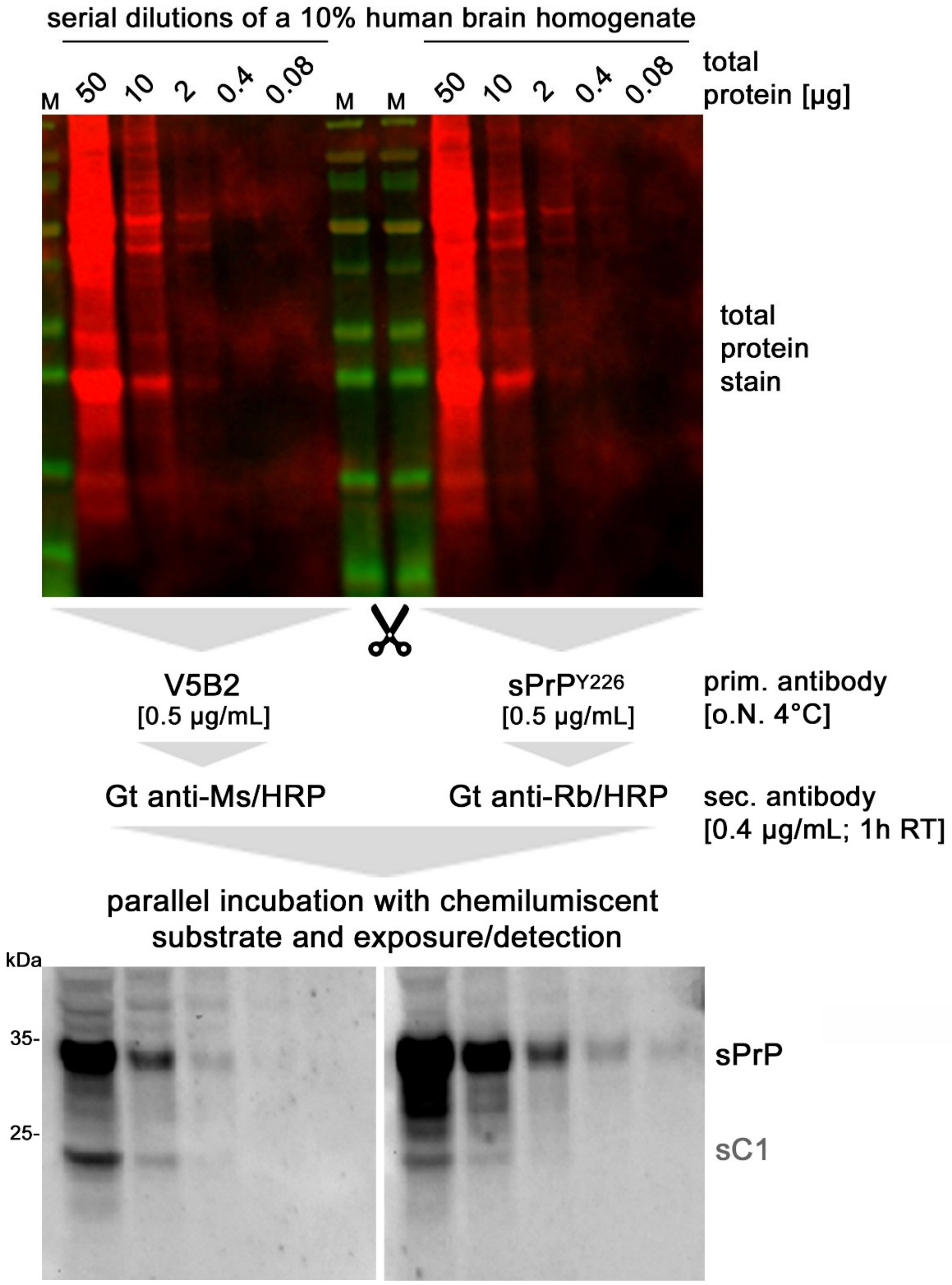

Supplementary Figure 3 (.jpg) Immunoblot analysis directly comparing monoclonal V5B2 and polyclonal $sPrP^{Y226}$ antibodies with regard to detection sensitivity towards denatured sPrP in serial dilutions of human brain homogenates. For ideal comparison, both blot parts derive from the same SDS gel and blotted membrane. After staining of total protein, the blot was cut into two parts (as indicated by the scissors symbol) for the sake of incubation with respective first and secondary (goat, Gt) antibodies (equal incubation times, equal antibody concentration, equal washing steps [as indicated]). After washing, both blot parts were re-united for incubation with the chemiluminescent substrate and for parallel detection. Shed PrP and the shed C1 fragment (sC1; resulting from shedding of already α-cleaved PrP) are detected by both antibodies with differing sensitivity. M = MW marker

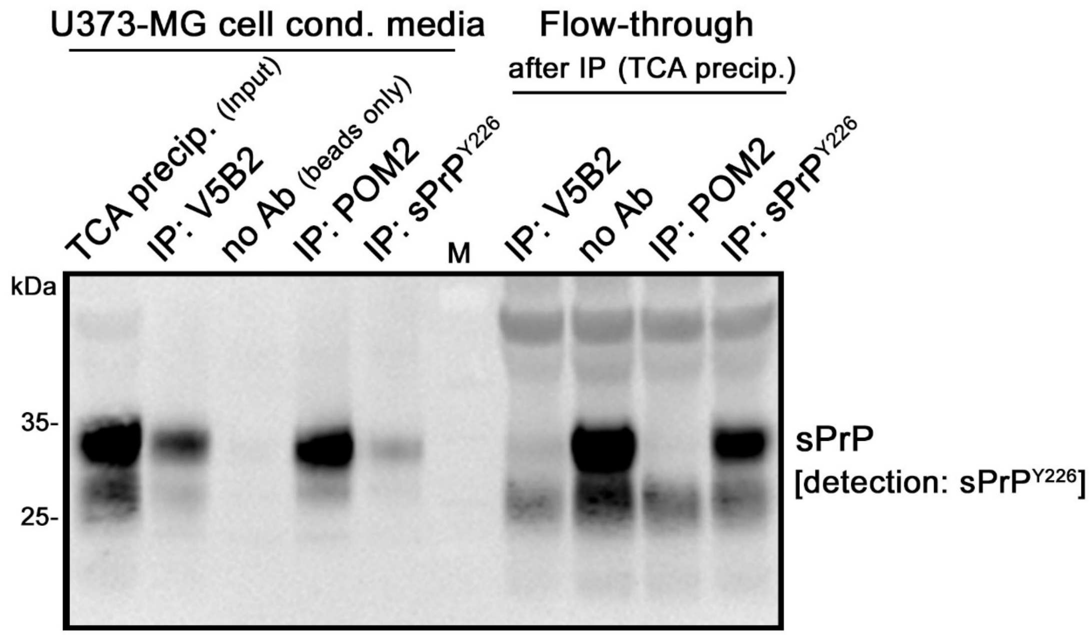

Supplementary Figure 4 (.jpg) Immunoprecipitation (IP) of released/shed PrP from conditioned media of human U373-MG cells. Efficiency of different antibodies for pull-down of shed PrP was in the (qualitative) rank order POM2 > V5B2 > sPrP^{Y226}. It should be noted that POM2 has four epitopes within PrP`s disordered N-terminal domain, which may support binding of two sPrP molecules per IgG. Moreover, POM2 could pull-down full-length PrP located on extracellular vesicles (and hence sPrP molecules possibly bound to the latter). TCA precipitated media (input) and flow-through (after IP; i.e. non-bound molecules) are shown for comparison. No unspecific binding was observed and, hence, no pull-down was achieved with beads only (“no Ab“). Detection of the blot was done with the polyclonal sPrP^{Y226} antibody

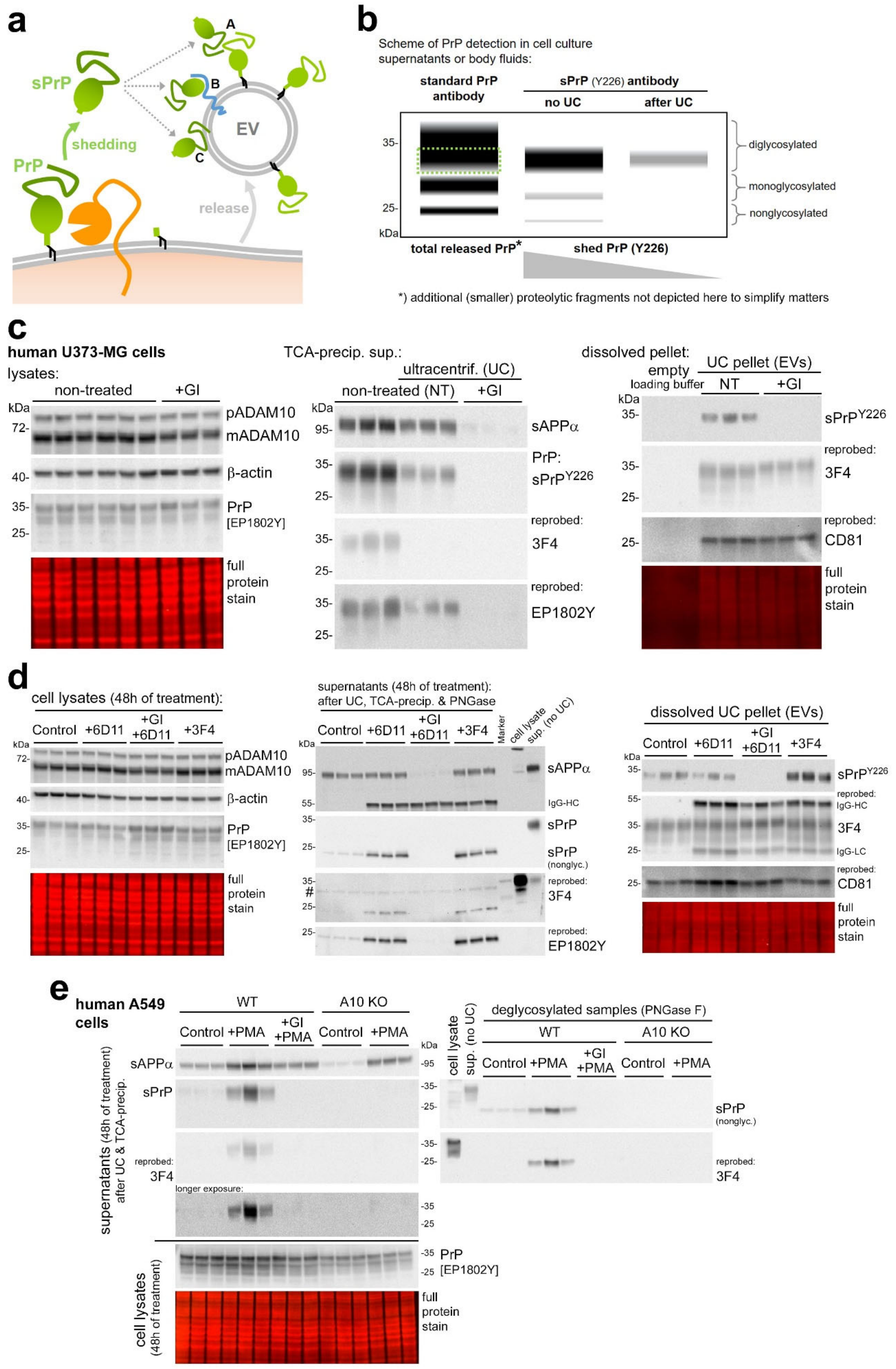

Supplementary Figure 5 (.jpg) (**a**) Model showing how sPrP, upon proteolytic release, secondarily binds to extracellular vesicles (EVs) in the extracellular space, body fluids or conditioned cell culture supernatants. This interaction is conceivable via homophilic interaction with

membrane-anchored PrP on the EV surface (option A), other receptors or binding partners for sPrP (option B) or association with the EV membrane or corona (option C), likely mediated by the flexible N-terminal tail. Depletion of EVs from a given sample (e.g., cell culture supernatants) would therefore lower amounts of sPrP. On the other hand, such a depletion needs to performed prior to assessment of sPrP with non-cleavage site-specific, classical PrP-directed antibodies, as it is usually masked in a biological sample by excessive amounts of full-length PrP (especially EV-PrP) as illustrated in the scheme (**b**). (**c**) Western blot analysis of lysates (left panel), conditioned media (with or without prior ultracentrifugation (UC); middle panel) and the dissolved pellet after UC (EV fraction; right panel) of U373-MG cells cultured for 48h confirms a substantial reduction in sPrP and PrP levels in supernatants after UC, while sPrP is detected in the respective UC pellet. Reprobing of the latter with 3F4 reveals all EV-associated PrP. Treatment with GI results in no detectable sPrP. CD81 is shown as a common marker for EVs. (**d**) Immunoblot assessment of untreated (control), antibody-treated (+6D11 or +3F4) or antibody/inhibitor co-treated (+GI +6D11) U373-MG cell lysates (left panel), their ultracentrifuged, precipitated and deglycosylated (PNGase) supernatants (middle panel), and respective UC pellets (right panel). sPrP^{Y226} antibody detects low amounts (note the reduction due to UC) of deglycosylated sPrP in controls (and glycosylated sPrP in a non-UC media control; middle panel, right lane). sPrP is increased upon treatment of cells with PrP-directed antibodies 6D11 and 3F4 and absent in co-treatment with GI. Immunoblot detection with 3F4 detects PrP in a control cell lysate and (albeit with weaker sensitivity) bands previously identified as PrPY226. Another pan-PrP antibody (EP1802Y) used for detection reveals a similar picture. (# indicates an unspecific band only detected with 3F4 in deglycosylated U373-MG media samples). (**e**) Similar analysis as in **d** but this time using A549 WT and ADAM KO cells (as in **Fig. 1d-f**) and PMA to stimulate ADAMs. Shed PrP (using sPrPY226 antibody) is barely detectable at basal conditions but increased upon PMA treatment in WT cells. No sPrP is detected in WT cells co-treated with PMA and GI as well as in ADAM10 KO cells. Detection with 3F4 does not reveal any other bands than those identified as PrPY226, neither in non-deglycosylated (left upper panel) nor in deglycosylated supernatants (right panel)

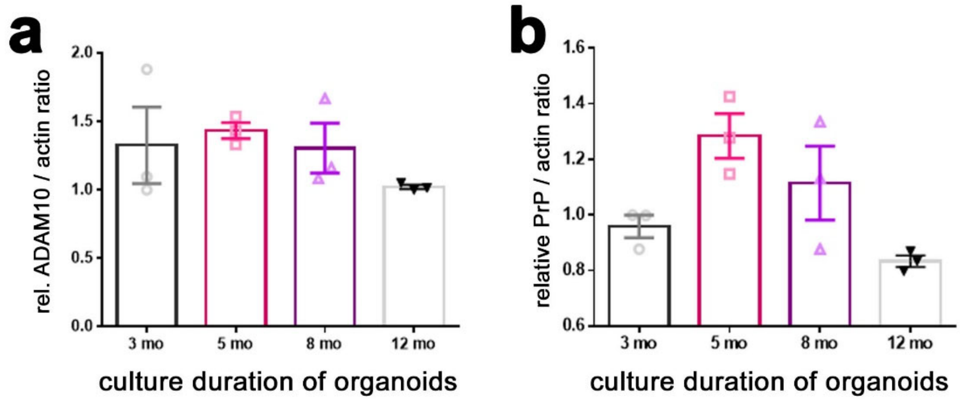

Supplementary Figure 6 (.jpg) Densitometric quantifications of the western blot assessment of ADAM10 (**a**) and PrP (**b**) protein levels in brain organoids at different maturation stages/periods in culture (related to data presented in **Fig. 4d**). The respective actin signal was used for normalization (n=3 organoids per time-point; mean ± SD)

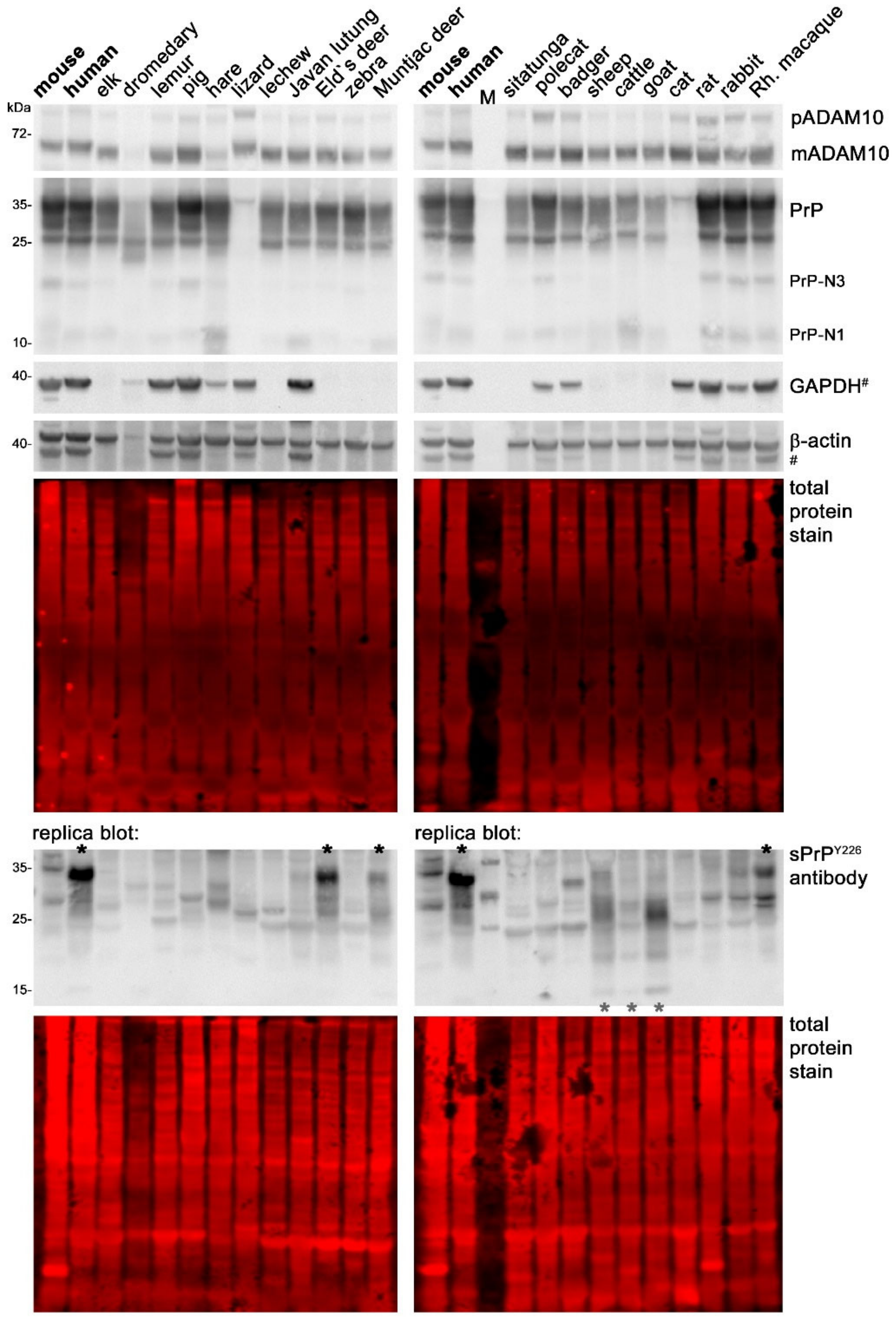

Supplementary Figure 7 (.jpg) Immunoblot analysis of CNS tissue samples of different animals. ADAM10, total PrP (including shorter N-terminal α- and γ-cleavage fragments N1 and N3, respectively), GAPDH and β-actin were detected on the upper blots, while sPrP was detected on a replica blot. Due to sequence/epitope differences, not all proteins are detected equally in all species. This especially was the case for GAPDH, which made us re-probing the blot with another housekeeping marker, β-actin (# indicates the previous GAPDH signals). The dromedary brain sample (4th lane) was pretty degraded at the time of assessment, and cat PrP could not be detected with the POM2 antibody used here (4th last lane). Importantly, while imperfect preservation and partial degradation of samples may be an issue here, and although unspecific bands appear upon detection with the polyclonal $sPrP^{Y226}$ antibody, a pattern similar to human sPrP appeared in the samples from Rhesus macaque as well as Eld`s and Muntjac deer (highlighted by black asterisks at the top of the blot); and a pattern reminiscent of a shed C1 fragment (see Supplementary Figure 3) was observed in sheep, cattle and goat brain (grey asterisks at the bottom of the blot). M = MW marker

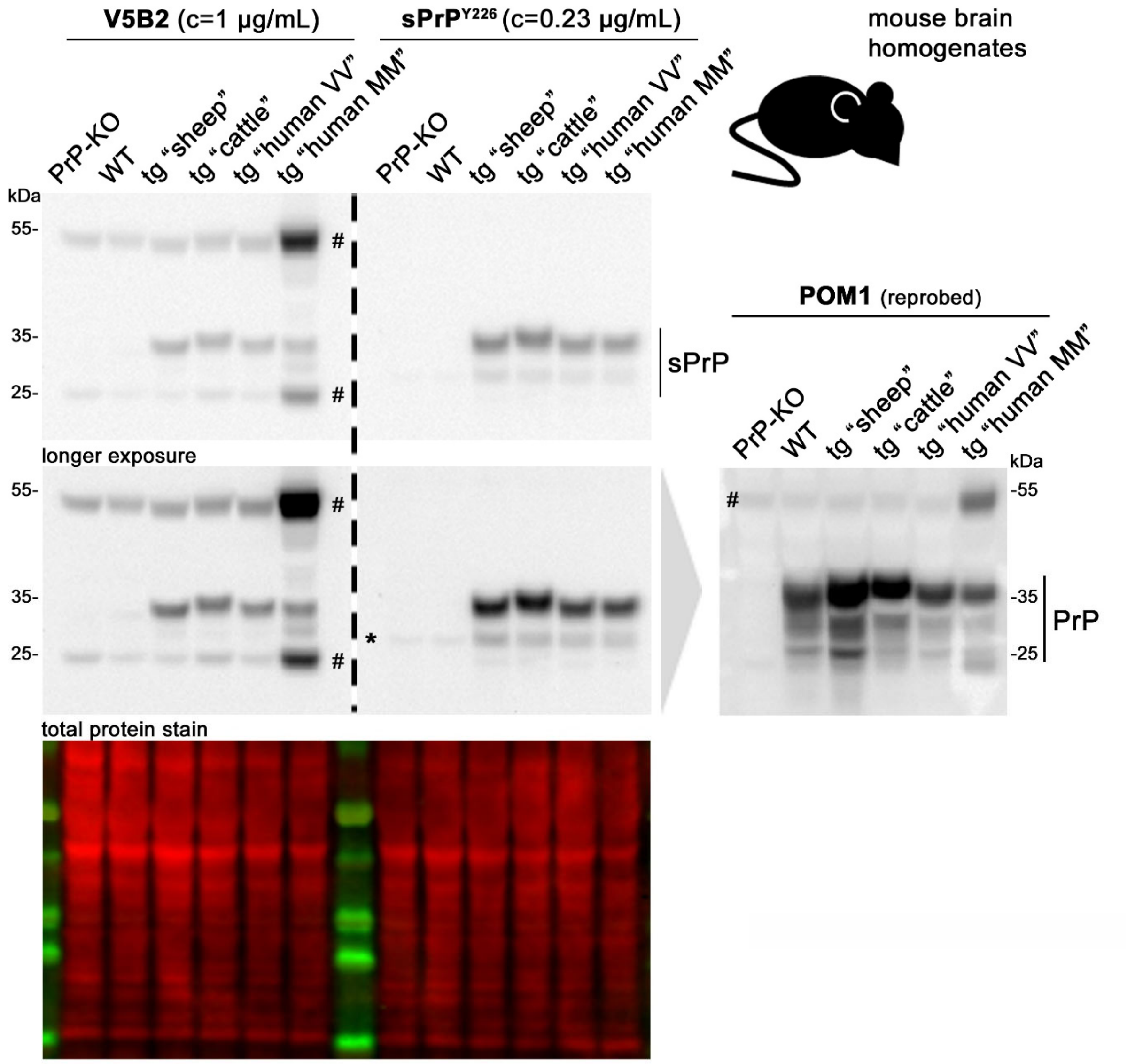

Supplementary Figure 8 (.jpg) Comparison of monoclonal V5B2 and polyclonal sPrP^{Y226} in immunoblot analysis of mouse brains. Duplicate samples were run in one gel and blotted on one membrane (see total protein stain) and only separated (dashed line) for incubation with indicated primary (and respective secondary) antibodies. After washing, both blot parts were handled in parallel for chemiluminescent substrate incubation and detection. Both antibodies detected shed PrP only in transgenic (tg) mice expressing sheep, cattle or human PrP (for the latter, two different lines with MM or VV polymorphism at PrP position 129 were used). No specific signals were detected in PrP-KO or WT mice. Despite very similar overall results, polyclonal sPrP^{Y226} revealed stronger specific signals albeit a lower antibody concentration. While detection with mouse antibodies V5B2 and (to a lesser extent) POM1 (used for re-probing/detection of total PrP) and their respective anti-mouse secondary antibodies revealed bands for IgG heavy and light chains present in the samples (indicated by #), sPrP^{Y226} showed a weak PrP-independent unspecific band in PrP-KO and WT brain (running at the height of monoglycosylated sPrP in tg mice; marked by an asterisk)

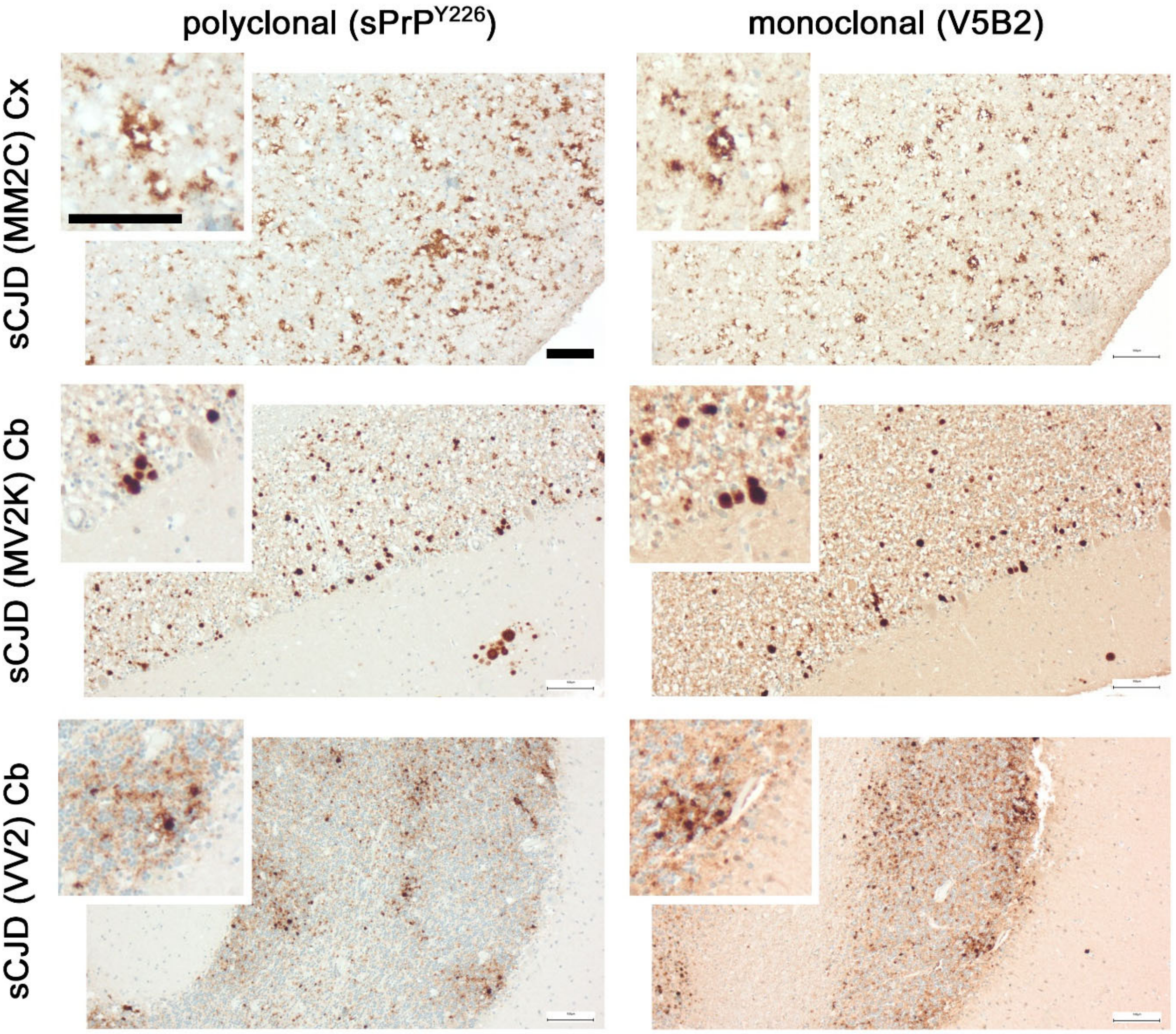

Supplementary Figure 9 (.jpg) Comparison of polyclonal sPrP^{Y226} and monoclonal V5B2 antibody in immunohistochemical assessment of brain sections of three different cases of sporadic CJD (subtype classification as indicated on the left). No PK digestion has been performed here. Cx = cortex, Cb = cerebellum. Scale bars: 100 µm

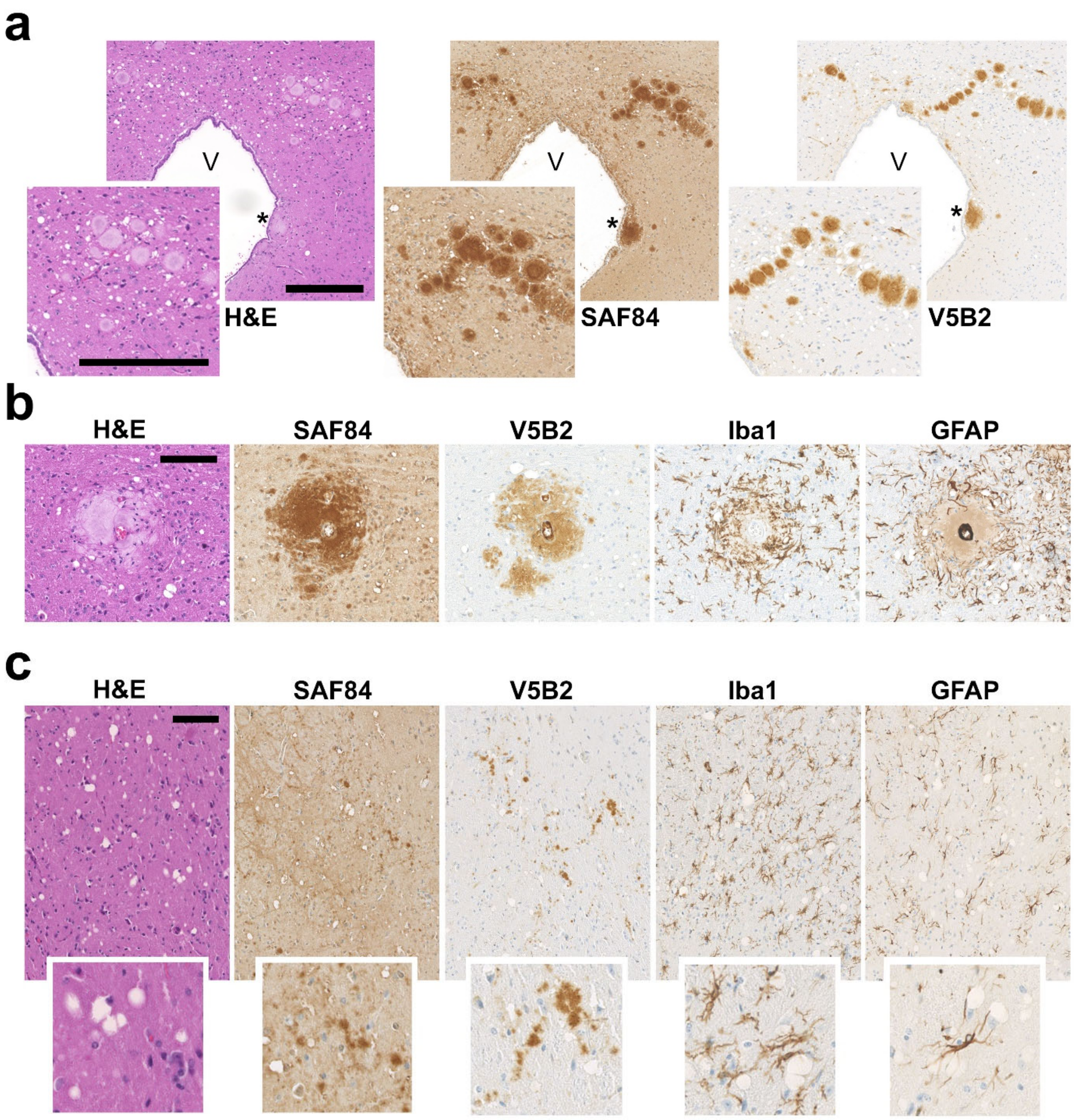

Supplementary Figure 10 (.jpg) Immunohistochemical analyses of prion-infected transgenic mice. (**a**,**b**) Tg338 mice (expressing ovine PrP) infected with NPU1 prions show extended clusters of large prion deposits in the brain stem. Shed PrP (detected with V5B2 antibody) associates with many, yet not all of these deposits. Asterisk highlights a sPrP-positive deposit close to the ventricle (V) wall. (**b**) A representative large and amyloid-like prion deposit around a brain vessel (in the center) is positive for sPrP and surrounded by activated glia (Iba1: microglia; GFAP: astrocytes). (**c**) Prion deposits, distribution of sPrP and activated glia cells in a subthalamic area of vCJD-infected transgenic mice expressing bovine PrP. H&E staining in **a**-**c** also reveals spongiform changes. Scale bars: 250 µm (in a), 100 µm (in b,c)

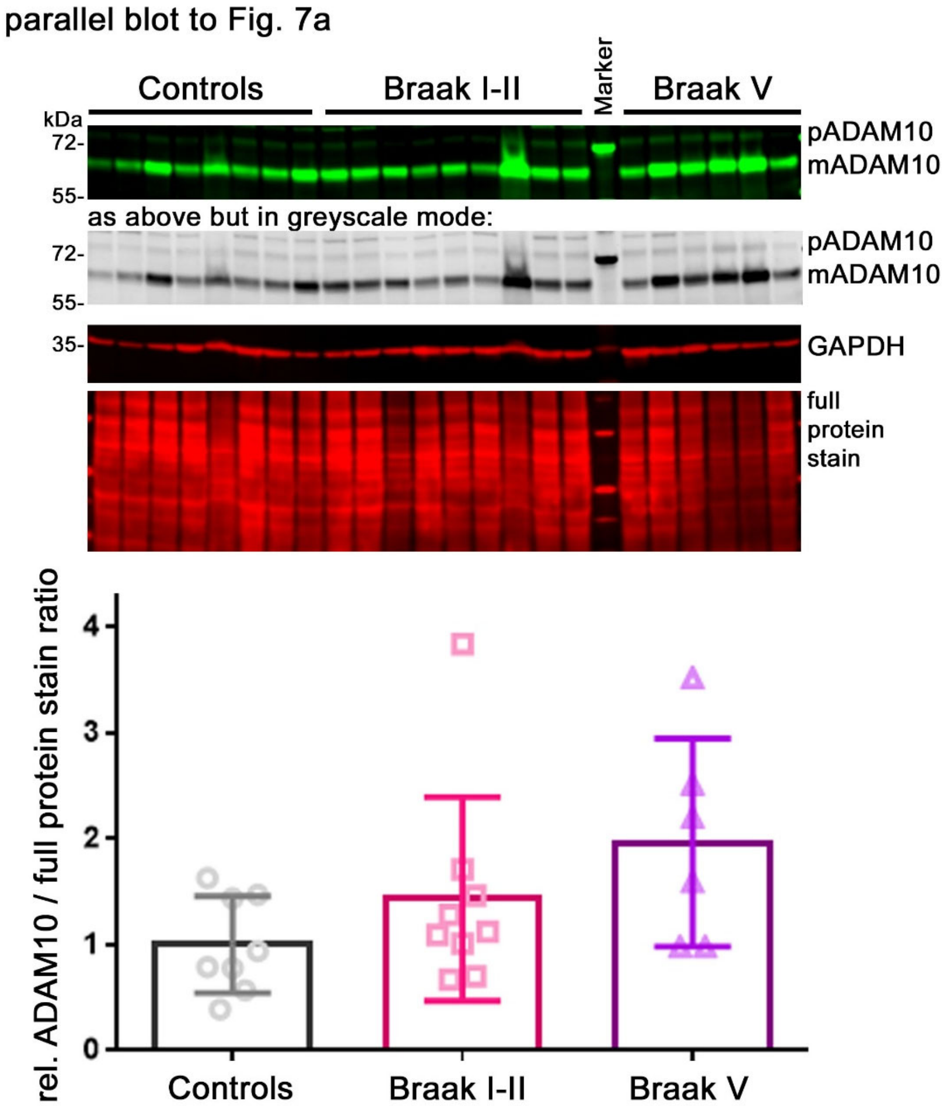

Supplementary Figure 11 (.jpg) Immunoblot assessment of ADAM10 (green fluorescence signals; greyscale mode better reveals a rather weak band in these samples for premature ADAM10) of control [n=8] and AD brain samples (Braak I-II [n=9], Braak V stages [n=6]) presented in **Fig. 7a**. (GAPDH is shown as a housekeeping gene product and full protein stain as loading control). Quantification of fluorescent signals (normalized for respective full protein stain signal) suggests a moderate increase in ADAM10 protein levels with advanced disease stage (controls set to 1, mean ± SD)

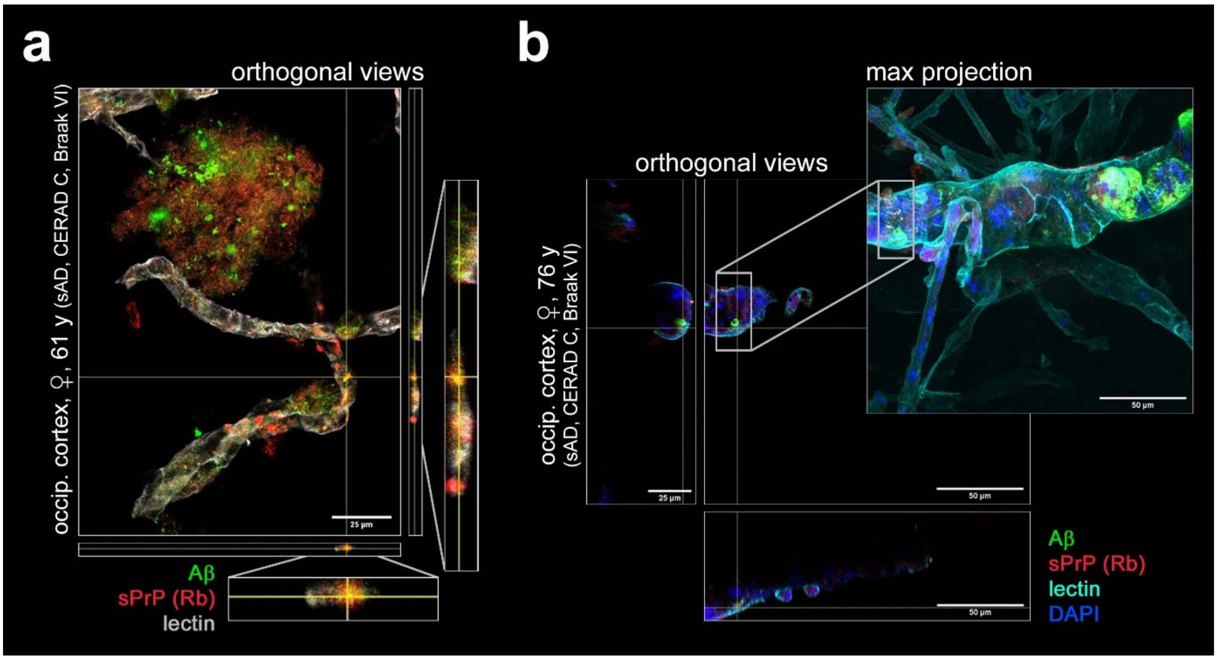

Supplementary Figure 12 (.jpg) Confocal IF microscopy of brain vessels isolated from human AD brain. (**a**) The same sample/analysis as in Fig. 7d, yet provided as orthogonal view representation highlighting the colocalization of sPrP (detected by polyclonal $sPrP^{Y226}$; Rb) and amyloid (Aβ) in/at brain vessels. (**b**) Orthogonal views and max projection of sPrP and Aβ in purified brain vessel of another AD patient (same as in Fig. 7e). DAPI was used to stain nuclei, lectin as an endothelial marker. Scale bars as indicated
